# Experimental Study on the Mechanical Properties and Microstructures of Cenosphere Concrete

**DOI:** 10.3390/ma16093518

**Published:** 2023-05-04

**Authors:** Krishna Prakash Arunachalam, Siva Avudaiappan, Erick I. Saavedra Flores, Pablo Fernando Parra

**Affiliations:** 1 Department of Civil Engineering, University College of Engineering Nagercoil, Anna University, Nagercoil 629004, India; 2 Departamento de Ingeniería Civil, Universidad de Concepción, Concepción 4030000, Chile; 3 Centro Nacional de Excelencia para la Industria de la Madera (CENAMAD), Pontificia Universidad Católica de Chile, Av. Vicuña Mackenna 4860, Santiago 1030000, Chile; 4 Department of Physiology, Saveetha Dental College and Hospitals, SIMATS, Chennai 600077, India; 5 Departamento de Ingeniería en Obras Civiles, Universidad de Santiago de Chile, Av. Ecuador 3659, Estación Central, Santiago 9170022, Chile; 6 Faculty of Engineering and Sciences, Universidad Adolfo Ibáñez, Santiago 7941169, Chile

**Keywords:** cement-OPC, silica fume, cenosphere, SEM, FTIR

## Abstract

The most valuable components of coal fly ash are cenospheres. Cenospheres are hollow spherical particles produced during the coal-burning processes. As a result of their excellent characteristics, such as high workability, high heat resistance, low bulk density, and high strength, cenospheres can be used in the manufacturing of lightweight cement concrete. The research efforts and outcomes are to produce long-lasting cement-based lightweight concrete (LWC) composites with good mechanical properties. The novelty of this investigation is to determine the cement concrete strength when silica fume (SF) and cenospheres (CS) were used as a replacement for cement. Throughout the experiments, a consistent substitution of 12% silica fume was incorporated into cement mass. Silica is used as a micro filler and pozzolanic reactant to strengthen concrete. The concrete mixtures were tested to ensure they met the requirements of the lightweight concrete in terms of their mechanical, physical, and durability qualities. According to the findings, lightweight concrete standards were met, and environmental sustainability was improved with the use of these mix proportions. Concrete specimen’s self-weight decreases by 35% with 30% cenosphere as a replacement. The micrograph shows the lack of portlandite is filled by mullite and other alumino silicates from the cenosphere. In order to achieve sustainability in concrete manufacturing, these mixtures can be suggested for the making of structural LWC that makes use of a large volume of industrial waste while conserving cement and natural resources.

## 1. Introduction

The usage of lightweight concrete (LWC) in structural applications has been around for a long time. In the past twenty years, there has been a significant amount of development in the field of lightweight concrete. Cenospheres, also known as CSs, are often produced as a byproduct of the burning of coal in thermal power plants. These cenospheres are hollow, lightweight spheres that are composed largely of silica and alumina and are filled with air or an inert gas. CS can be used as structural lightweight filler. CSs vary in appearance from grey to practically white, and their density is between 0.4 and 0.8 g per cubic centimeter. CS is a non-toxic, non-flammable, non-abrasive, light, waterproof, and insulating material, and so they can be used in a many applications and can be used as fillers [1,2,3,4,5,6]. Low-density concrete can be produced using CS fillers. Fly ash is one of the components that make up the CS.

The CS is used often in LWC intially, and it is later used in ultra-lightweight cement (ULWC), which is a type of innovative cementitious material that is now being researched. CS is a cementitious material, and concrete incorporated with CS has higher mechanical strength of at least 60 MPa and a low density of at least 1450 kg/m [7,8,9]. The performance of LWC and ULWC that make use of CSs might vary greatly due to the ratio of replacement, the type of binder used, the type of admixture, curing temperature, and the qualities of the CS [10,11,12,13]. The utilization of ULWC/LWC that makes use of CS has gained a lot of interest due to the fact that it has low density and high heat conductivity.

Experiments that were carried out on either LWC or ULWC employ other forms of lightweight aggregate (LWA), such as expanded polystyrene, thermal ash aggregate, leca, expanded glass aggregates, and natural fibers, which have been reported previously [14,15,16,17]. Wu et al. [18] experimented on two distinct types of CS that were of a different sizes in order to test the mechanical properties of CS of varying size and its thermal conductivity. An investigation was carried out by Huiskes [19] on a ULCC geopolymer made up of 70% fly ash and 30 percent slag. This geopolymer is a combination of lightweight expanded glass particles of varying sizes. They examined how the size of the LWA particles affected the mechanical qualities as well as the thermal conductivity of the material. Yan et al. [20] investigated the effects of the reinforcement bending ratio, shear span, fraction of fiber volume, section depth on the failure mode, punching shear resistance, and load–deformation behavior of flat slabs made using OPC and CS-based fiber-reinforced ULWC flat slab. CS was combined with LWA concrete and ULWC composite by Sohel et al. [21], who then studied flexural fatigue behavior and developed a mathematical derivation to determine fatigue strength. Wang et al. [22] evaluated cement paste thickness to determine CS distance in OPC-based ULCC. In order to determine exactly the worth of a CPT, they developed an innovative method that was based on geometric correlations. An experiment on the mechanical characteristics of ULCC was carried out by Liu et al. [23] utilizing CS when it is cold, between 0 and 60 degrees Celsius. The effect that temperature has on ultimate strength, stress–strain curves, Poisson’s ratio, modulus of elasticity, and tensile behavior was investigated using these methods. The author Huang et al. [24] investigated the effect that temperatures as high as 1000 degrees Celsius had on the mechanical properties of ULCC. The experiment investigated the behavior of the stress–strain curve, as well as the residual compressive strength, the modulus of elasticity, the weight loss, and the failure mechanism. At a range of temperatures, the specimen that had been heated was examined using both a macroscope and a microscope to search for signs of physical deterioration, such as a change in color, cracking, or fracture. Wang et al. conducted research on the mechanical characteristics of ultra-low-carbon steel that was reinforced with steel fibers [25,26]. CS was used by Liu et al. [27] in order to carry out an experiment involving the internal curing of OPC-based cement. According to their experimental results, curing decreased the amount of autogenous shrinkage that the cement concrete experienced and enhanced its compressive strength. A composite model was used by Rheinheimer et al. [28] to estimate the thermal conductivity of the CS on the basis of the results of the ULCC porosity and thermal conductivity tests conducted on the specimens. According to Hanif et al., the addition of CS to cement-based fiber-reinforced composites would provide a feasible material capable of manufacturing lightweight and strong structural components for building that would stimulate viable development [29]. The use of ULCC/ULWC/LWC combined along with CS is steadily expanding due to the materials’ low weight and excellent resistance to thermal conductivity, as well as their enhanced insulating performance and tolerance to high temperatures [30,31,32,33]. As a direct consequence of this, studies are now being carried out in a variety of contexts to investigate the durability of composite materials and the functional enhancements they provide. The CS is a multipurpose construction material that may be used for a wide variety of projects. Cenosphere and fly ash were used as a replacement for Ordinary Portland Cement (OPC) in high-performance mortar by Aamar et al. [34]. The effects on mechanical and durability properties were evaluated with different curing conditions and temperatures. Results showed that water curing at a higher temperature was effective, and CS incorporation (10–15%) improved flexural strength, fire resistance, acid attack, and water absorption. A study by Gupta et al. [35] investigated the use of rice husk biochar (RHB) in cenosphere-based mortar. The addition of RHB did not affect fresh properties and hydration kinetics, but improved water tightness and resistance to thermal damage at 450 °C. The material mix proposed can significantly reduce dead load of building members while maintaining sufficient strength for structural applications, offering sustainability benefits. A study by Salim et al. [36] investigated the use of cenospheres and waste glass powder as a replacement for cement in high-performance mortars. The combined effect of CS-WGP was examined at different curing conditions and temperatures. Results showed that water curing at elevated temperature was efficient, and a 10% replacement of CS-WGP showed improved rheological, mechanical, and durability properties.

Researchers have investigated CS mechanical qualities in concrete and in other materials. Little research has been conducted on adding CS to cement, so there is a need to study its high-performance effects by adding admixtures such as silica fume. The novelty of using cenospheres (CS) in concrete is that it provides a sustainable solution to the waste generated during the coal-burning process while also improving the mechanical and physical properties of the resulting lightweight concrete (LWC). This study also highlights the potential of combining CS with silica fume (SF) as a partial substitute for cement, which further enhances the strength and durability of the concrete. The micrograph analysis shows that the CS filling the lack of portlandite results in the formation of mullite and other alumino silicates, indicating that the CS can also contribute to the pozzolanic reaction and increase the cement hydration. Overall, this research offers a promising approach to producing high-quality LWC that meets the lightweight concrete standards, reduces the self-weight of the concrete, and promotes environmental sustainability by utilizing industrial waste materials.

The key findings of the study were that the replacement of cement with CS in LWC reduced the self-weight of the specimens by up to 40%, and the lowest water absorption was observed in LWC with 30% CS replacement. However, the compressive and splitting tensile strength decreased with increasing percentages of CS replacement beyond 30%. Additionally, the flexural strength of LWC with 15% CS replacement was found to be good, but it decreased with higher percentages of CS replacement due to the formation of internal voids and capillary channels in LWC. The study also revealed that the microstructure of LWC with CS and SF as partial cement replacements was dense, and the bonding between the materials was maintained. Moreover, the addition of CS to LWC led to the formation of mullite and other alumino silicate substances that compensated for the voids formed by the absence of portlandite. However, the microstructure of LWC with 30% CS replacement was found to be fully degraded, with visible cracks. Overall, the study contributes to advancing the field of cenosphere concrete by providing insights into the use of CS as a partial cement replacement in LWC, the impact of varying percentages of CS on the properties of LWC, and the microstructural changes that occur with CS and SF as partial cement replacements. The findings of this study can aid in the development of more sustainable and lightweight concrete mixes for construction applications.

## 2. Cenosphere Characterization

Cenospheres are a crucial component in the development of high-strength concrete, and their effectiveness depends largely on their chemical and phase composition, as well as their physical characteristics, such as particle size and shell thickness. For applications that require maximum strength, particle thickness becomes particularly important, as particles with thicker shells provide greater strength compared with those with thinner shells [37,38].

Cenospheres derive their characteristic chemical makeup from the coal with which they are burned, as well as the coal itself [39,40,41,42,43]. The major components of cenospheres include Fe, Ca, Mg, K, LOI, and aluminosilicate, along with smaller amounts of Ti, Na, P, S, and inorganic compounds. Due to the non-uniform distribution of mineral impurities in burned coal, cenospheres exhibit a broad chemical compositional range. Additionally, chemicals are often added to coal before burning to enhance its combustion efficiency. Cenospheres are exclusively found in the Ferrosialic and Sialic groups, with the development of internal gas occurring when the molten ash droplet contains at least 5% iron oxide [44]. However, studies by Goodarzi et al. [44] have shown that many cenosphere specimens contain even less iron oxide, contradicting the conclusion that iron is always necessary for cenosphere development [45,46,47,48,49]. Ferrocalsialic cenospheres exhibit magnetic properties, while Sialic cenospheres do not [50]. Fly ash exhibits a chemical makeup similar to that of cenospheres, with the exception that cenospheres contain more carbon, as seen by the greater LOI of fly ash [51,52,53,54].

## 3. Experimental Program

### 3.1. Materials

#### 3.1.1. Cement

**Figure 1:**
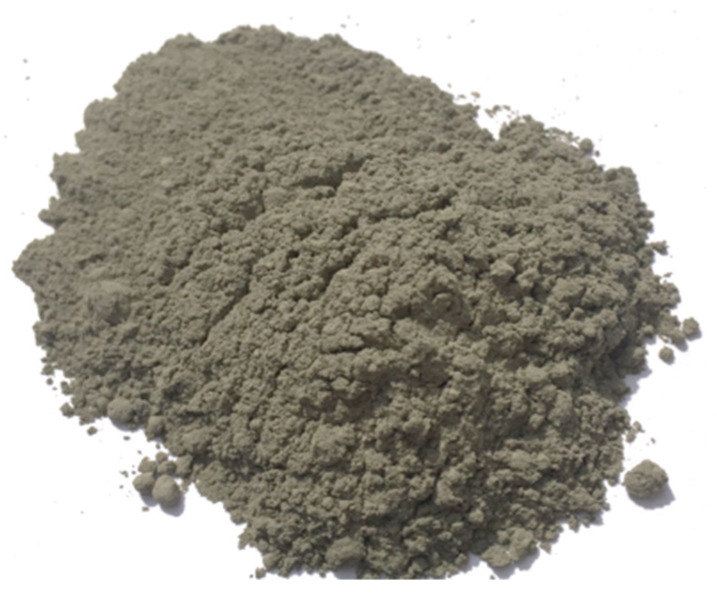
Cement.

The cement used in this study was Type III (high early strength and low alkali) as stated in BS EN 197-1 [28] (Figure 1). Standard concretes were formed using 42.5-grade cement according to the ASTM code. Table 1 shows the chemical composition of the cement utilised in this investigation.

#### 3.1.2. Silica Fume

**Figure 2:**
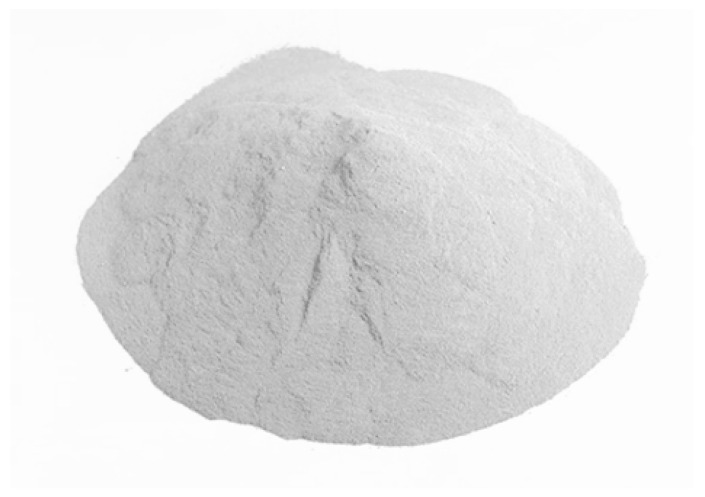
Silica Fume.

Chemical composition of silica fume is shown in Table 2. Silica fume was gathered from a local source (Astra Chemicals, Chennai, India) and met the standards of BS EN 13263-1:2005 [29]. Processing requirements, drying conditioning requirements, fineness, and ignition loss requirements are among these criteria. The silica fume used had particles that ranged in size from 0.3 mm to 0.075 mm (Figure 2).

#### 3.1.3. Cenosphere (CS)

**Figure 3:**
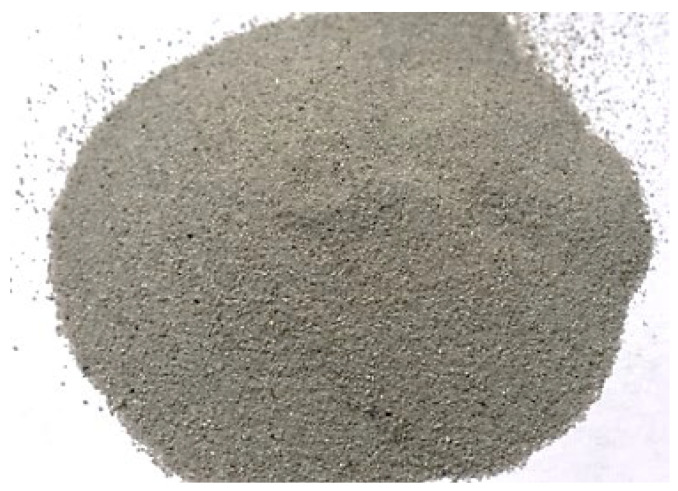
Cenosphere.

**Figure 4:**
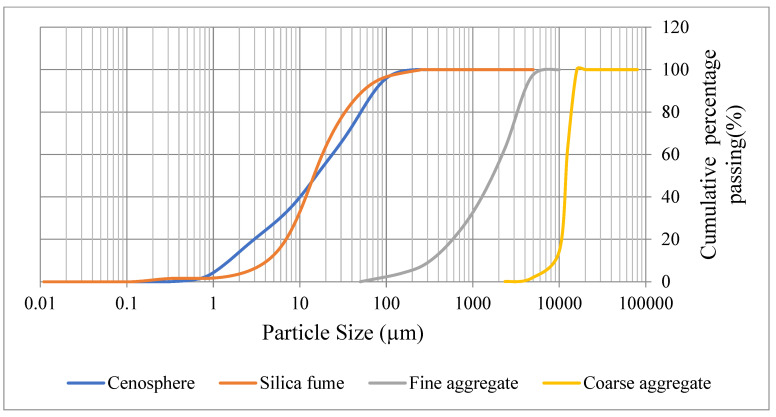
Particle size distribution of Cenosphere, Silica Fume, and Fine and Coarse aggregate.

CS were obtained from Astra Chemicals, Chennai, India. Regarding particle size, CS grade 300 has the largest particles at 300 um (Figure 3). The chemical content of the CS utilized is shown in Table 1. The values of physical and chemical characteristics of the CS employed in this investigation (such as bulk density, moisture content, and pH) are listed in Table 3 and Table 4. Particle size distribution of cenosphere and silica fume is shown in Figure 4.

#### 3.1.4. Sand

Crushed stone sand was utilized in this investigation in accordance with BS EN 12,620 [30] standards. A saturated surface dry (SSD) sand was employed according to ASTM C566 [31]. As seen in Figure 4, the aggregates were sieved to achieve the appropriate particle size distribution per ASTM D6913/D6913M-17 [32] standard standards.

## 4. Experimental Testing Methods

### Mix Proportions and Casting of Specimens

The concrete cubes were made using a cement: coarse aggregate:fine aggregate ratio of 1:1:2 and a water/binder ratio of 0.45; the concretes were made using concrete mixing machines with this proportion. The CS was utilized to replace cement 30% by weight, and the replacement details are shown in Table 5 and Table 6. To improve the strength of the specimens, the basic mix proportions were adjusted by employing 12 percent (40.32 kg/m^3^) silica fume as a partial substitute for cement as a constant replacement in all specimens [43]. ASTM 305-14 [55] was followed in the preparation of all concrete mixtures. A mechanised concrete mixer was used to make each of the mixes. Before being put to use, the fine aggregates, together with the binders (CS, FA, and cement) are mixed dry for two to three minutes. Wet mixing was carried out on the mix for roughly 14–15 min, split into two periods. Following the addition of two-thirds of the water and the superplasticizer to the dry mix and the subsequent mixing for approximately 5 min, the remaining superplasticizer and water were added, and the mix was mixed an additional time. After the mixing procedure was finished, the concrete mixtures were poured into steel cubes of size 50 mm × 50 mm × 50 mm and prisms of size 25 mm × 25 mm × 300 mm. The cubes were used to test the concrete’s compressive strength, water absorption, fire resistance, and acid attack, while the prisms were used to test the concrete’s flexural strength. The cube specimens were put through compressive strength testing, acid attack testing, and water absorption testing. For the purpose of flexural strength, prisms were cast. The casting molds that were used (prisms and cubes) were all made of steel and lubricated on the inside to prevent the creation of voids and to make demolding simpler. All of the casting molds were used. A tamping rod with a diameter of 12 mm compacted each layer thoroughly. After compaction, the upper layer was flattened with a trowel and allowed to dry for 24 h at 31 °C and 75% humidity. The concrete cubes were stored in a curing tank, to which potable water was added the following day. The temperature was kept at 30 degrees Celsius, and humidity was 60 percent. In accordance with ASTM C39 [56], the specimens were evaluated with the help of a hydraulic compression testing machine that had a capacity of 2000 kN.

## 5. Results and Discussions

### 5.1. Workability

**Figure 5:**
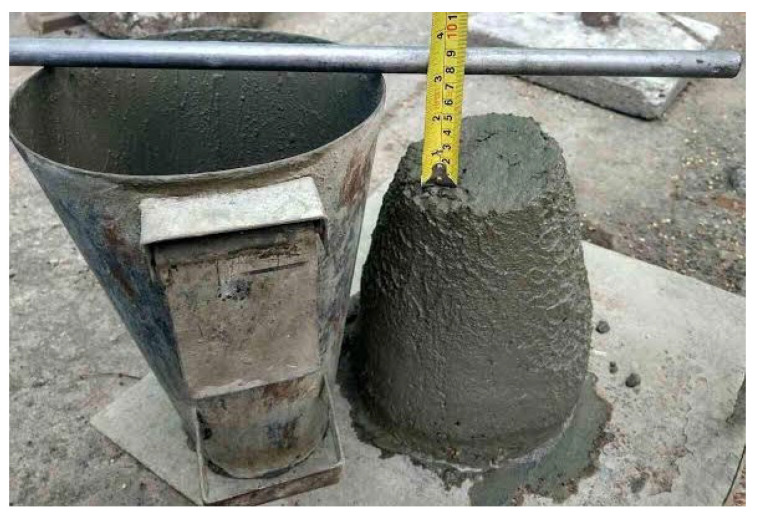
Workability Slump Test.

The slump values of concrete mixes were prepared and tested. The results showed that when the percentage replacement of CS with continual replacement of silica fume increased, the workability was reduced. The value ranged from 100 mm to 65 mm, ensuring medium workability throughout all mix proportions. All of the mix proportions demonstrated true slump behavior. Slump values for CS5, CS10, CS15, CS20, CS25, and CS30 were 10 percent, 20 percent, 30 percent, and 35 percent lower, respectively. The lowest slump value in CS30 specimens was 65 mm, while the highest slump value in CS5 and control specimens was 100 mm. It was discovered that when the CS increased, the workability decreased. Figure 5 shows the Slump Cone test.

### 5.2. Compressive Strength

**Figure 6:**
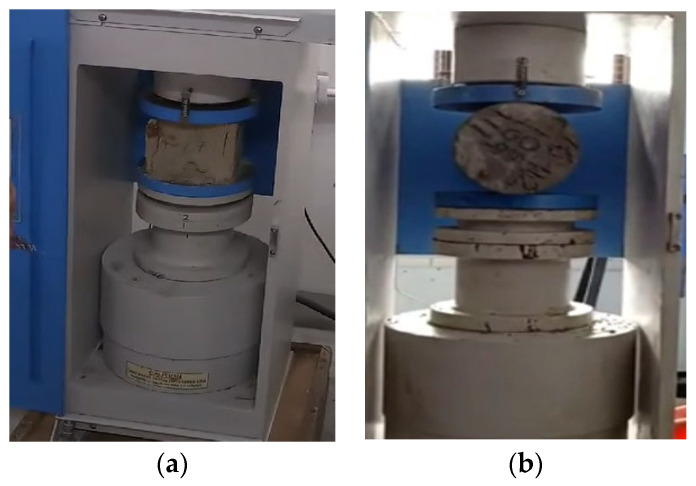
(**a**) Compression Test and (**b**) Split Tensile Test.

**Figure 7:**
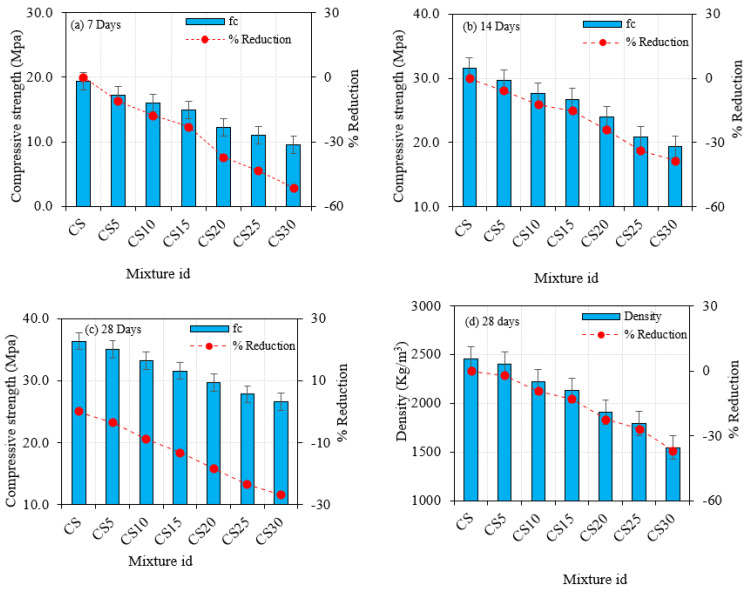
Concrete compressive strength at 7, 14, and 28 days and density with CS.

The capacity to carry weight is determined by compressive strength, which is an essential feature of CBM. The compression testing equipment was used to investigate the behavior of cement concrete substituted with SF and CS when subjected to compressive pressure. ASTM C109/C109M-20a [57] was used to conduct the compressive strength test. The strength of the concrete cubes was examined after 7 days, 14 days, and 28 days, and the findings are shown in Figure 6a and Figure 7 and Table 6. The findings demonstrate that adding CS to concrete diminished its mechanical strength; nevertheless, the strength may be sustained by utilizing silica fume as a constant replacement in cement. With 30% CS substitution, the compressive strength almost exceeded 25 MPa; therefore, further addition was avoided. CS20 had the designed compressive strength at all ages, whereas CS30 had the lowest strength. In comparison with CS, the strength of CS5, CS10, CS15, CS20, CS25, and CS30 dropped by 3.60 percent, 8.80 percent, 13.11 percent, 18.31 percent, 23.50 percent, and 26.88 percent, respectively, after 28 days. This indicates the active CS material used in cement as a substitute slows strength acquisition, perhaps reducing microstructural cracks and shrinking. The density was also found to decrease [1,13].

### 5.3. Split Tensile Strength

**Figure 8:**
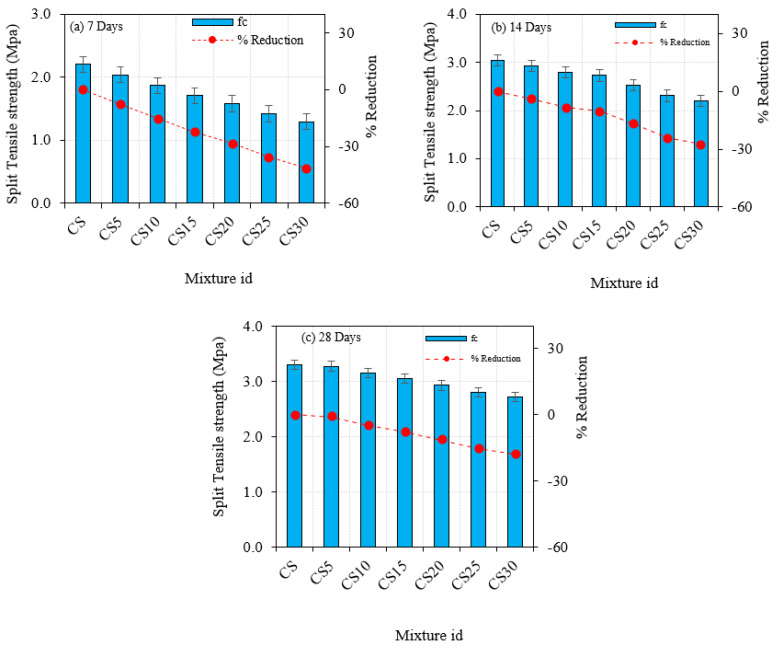
Concrete split tensile strength at 7, 14, and 28 days with CS.

The method of evaluating the tensile strength of concrete specimens that split across the vertical cross-sectional area is known as splitting tensile strength. The effect of variable CS substitution as a cement replacement material and constant silica fume on splitting tensile strength was examined. The results revealed that, like compressive strength, the average splitting tensile strength of silica fume and CS admixed concrete specimens diminished. All CS specimens’ splitting tensile strength declined with age. The splitting tensile strength achieved on concrete cylinders after 7 days, 14 days, and 28 days was experimentally examined for three specimens at each age, and the average findings were taken. Findings are presented in Figure 8, and the testing images are seen in Figure 6b. For CS5, CS10, CS15, CS20, CS25, and CS30, the splitting tensile strength decreased by 0.95%, 4.77%, 7.62%, 11.43%, 15.24%, and 17.78% after 28 days, respectively.

### 5.4. Flexural Strength

**Figure 9:**
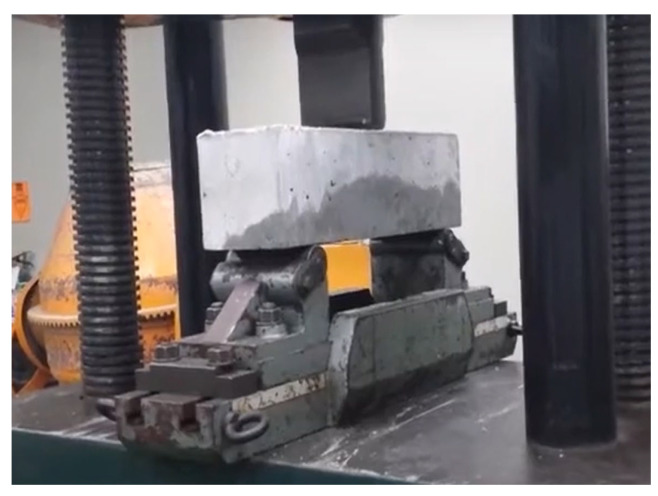
Flexural Testing.

**Figure 10:**
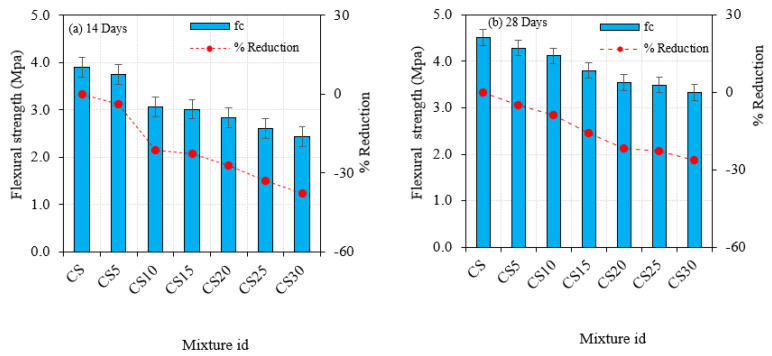
Concrete flexural strength at 14 and 28 days with CS.

The capacity of cementitious composites to withstand bending stresses is known as flexural strength. This test also determines the strength of the binding between the concrete elements. This test was carried out in compliance with ASTM C348-20 [58] requirements. A flexural testing machine was used to investigate the cement concrete behavior substituted with SF and CS under three-point loading. The growth of internal voids and capillary channels in the concrete reduced the flexural strength of CS, resulting in a deterioration in the quality of the concrete. The bonding capabilities of CS had a significant impact on the flexural strength of concrete, as an increase in the CS percentage impacted the fundamental strength of concrete. The CS replacement specimens developed strength as time passed, demonstrating the relative growth in mechanical strength with age. This finding demonstrates that flexural strength increases and decreases are similar to compressive strength increases and decreases. There was no discernible difference in behavior between CS admixed concrete and fibrous materials. As a result, the relativity of pozzolanic materials had an effect on enhancing mechanical strength but not on increasing flexural strength. The testing image is provided in Figure 9, and experimental findings are presented in Figure 10.

### 5.5. Water Absorption

Water absorption greatly influences the cementitious composite’s durability, and so water absorption is a crucial feature that needs to be assessed for composites based on cement, especially those that are immersed in water. The ASTM C1403-15 [59] requirements were followed for conducting the test. The specimens were dried in oven at 105 C for 72 h after they were demolded. The specimens are weighed after drying. After that, the specimens were immersed for 28 days in water. After 28 days, the specimens were weighed again to assess the amount of water absorbed. The following findings were drawn from the water absorption experiments and the observed findings. The continuous silica fume and changing CS replacement HPLWC demonstrated less water absorption than the control specimen, as shown in Table 5. When compared with the control specimen, the CS5, CS10, CS15, CS20, CS25, and CS30 demonstrated 16.7 percent, 19.35 percent, 33.72 percent, 30.72 percent, 22.42 percent, and 24.62 percent reductions in water absorption, respectively. When compared with CS0, the average water absorption in the CS15 specimen was just 1.14 percent, indicating a 36.49 percent drop in absorption. In comparison with CS0, the CS20 specimen exhibited a 27.82 percent reduction in water absorption. As a result of the findings, it was established that the inclusion of constant silica fume and changing CS reduced pores in high performance light weight concrete when compared with cement, and slightly increased pores when CS percentage surpasses 20%.

### 5.6. Acid Attack

**Figure 11:**
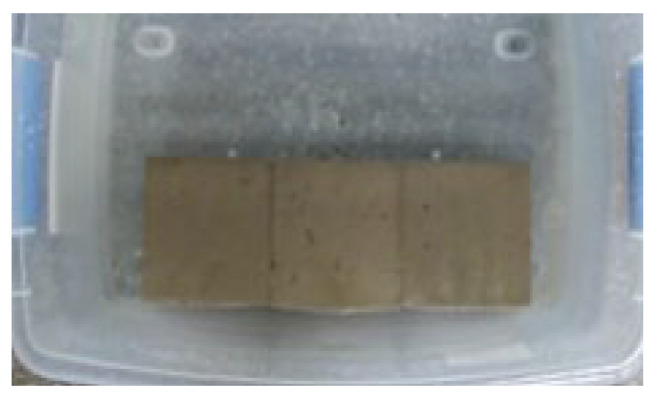
Specimens placed in sulfuric acid.

**Figure 12:**
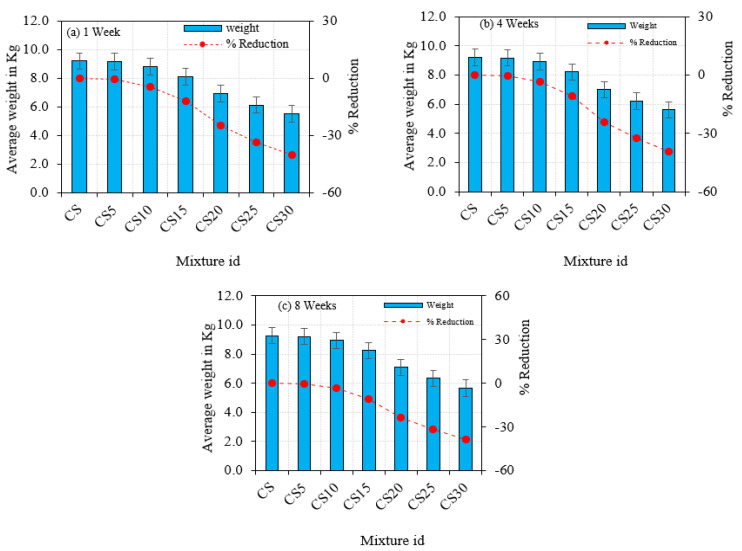
Acid Attack on CS Concrete.

The specimens were also put through an acid impact test to see how resistant they were to sulfuric acid. Owing to the unavailability of an applicable standard from either the ASTM or the BS, this experiment was conducted using a mix of several standards, such as ASTM C1012 and C563-07 [60,61], and from previous research [45,62]. The following technique was used for this test. A solution containing 5% sulfuric acid was made. The solution was changed every 10 days to retain the solution concentration. Before inserting the specimens in the solution, their weight was estimated. The specimens were immersed in the standard solution for three, seven, and twenty-eight days. The surfaces of the specimens to be investigated were thoroughly cleaned at the time of testing. Each specimen was tested for percent loss in weight and percent loss in strength on the test day. The test specimens’ visual appearance after being subjected to different periods was observed. After four weeks of exposure, there was no discernible difference in the visual appearance of the test specimens. Surface erosion, cracking, or spalling of the specimens was not obvious until 4 weeks. However, after 8 weeks, a small amount of surface erosion was observed. Testing images and findings are presented in Figure 11 and Figure 12. External fissures and bulging of the surface are visible as a result of the 8-week erosion. This bulging was caused by weight increase, which caused the internal structure to expand. Sulphonate production causes bulging, which increases density and weakens the structure. Parthiban et al. reported that regarding higher resistance to sulphuric acid on GPC concrete, the results were similar to the findings of CS concrete [63].

### 5.7. SEM and XRD Analysis

**Figure 13:**
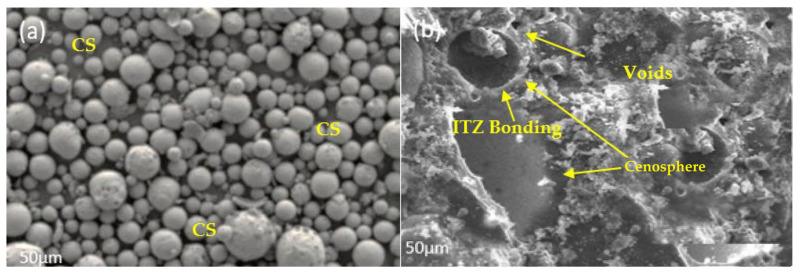

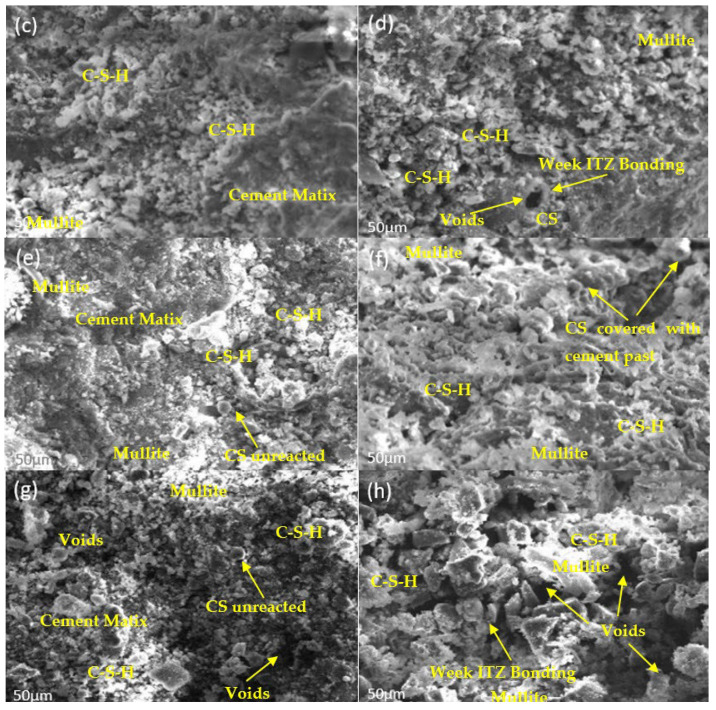
SEM Images of Cenosphere Concrete: (**a**) CS, (**b**) CS0, (**c**) CS5, (**d**) CS10, (**e**) CS15, (**f**) CS20, (**g**) CS25, and (**h**) CS30.

Scanning electron microscopy and XRD patterns were also used to examine the specimens. A SEM study was performed at an analytical focus of 5000×, with a high resolution of 3.5 nm and an energy of 20 keV. For this study, specimens of 10 mm cubes were trimmed by a saw cutter on the 28th day. The XRD analysis was performed using a Bruker AXS with Cu K-beta radiation and two scans with a scale factor of 0.02 and analysis time of 10.00 Deg/minute. After 28 days of water curing, samples were taken from the cubes and ground on ball mills which need to pass through a filter of size 90 μ. The micrograph of the CS0 specimen in Figure 13b reveals the density of the control specimen as well as hydration products. The behavior of the control group was as expected based on the literature. In the sample, there was a significant amount of calcium hydroxide production. Figure 13c depicts the clustered structure of the CS5 micrograph. The formation of the structure can be related to the existence of alumina silicate in the silica fume as well as CS. Ettrignite production was also discovered [14], and this result may be linked to a decrease in hydration, as was demonstrated by earlier research [15]. As can be seen in Figure 13d, the carbonation effect and pozzolanic reaction led to the absorption of calcium hydroxide, which led to the disintegration of the CS10 specimen. The reaction of silica fume, on the other hand, is what maintained the structural integrity [1]. This is because of the silicious properties that it possesses. Figure 13e is an image from a scanning electron microscope that depicts the CS15 specimen. On the basis of the observations made, it is evident that the presence of aluminosilicate and mullite, both of which were created by the addition of cenosphere, filled what was previously occupied by portlandite [16,17]. In addition, a number of microstructural fractures were found by the imaging. Figure 13f, which is a SEM image of CS20, shows that the CS begins to appear in abundance as a result of a higher percentage of CS replacement. This can be seen as evidenced by the abundance of CS. Analysis of the interfacial transition zone (ITZ) revealed how CS affects durability and mechanical characteristics. A distinct interface and fissures among cement paste and CS particles can be observed in Figure 13h, demonstrating a poor connection between mortar and unreacted CS elements, which explains the loss of compressive strength in CS mixes and the reason why employing CS in mortar weakens the ITZ. When CS was used as a fine aggregate substitute, Sudeep Kumar et al. [64] noted a similar phenomenon in terms of ITZ. According to Asad et al.’s [65] investigation on CS microstructural behavior, the permeable characteristics of the particulates and poor packing, particularly at a high level, led to a noticeable rise in matrix porosity as the percentage of CS arose. Despite the C-S-H produced by CS through pozzolanic reaction, the inner porous structure of the particles was revealed upon complete consumption of the CS shell, as seen in Figure 13b. This explains the greater water absorption capacity of mixtures that contained CS as compared with the control mixture quartz. These findings are consistent with previous reports of Parthiban et al. [66,67].

**Figure 14:**
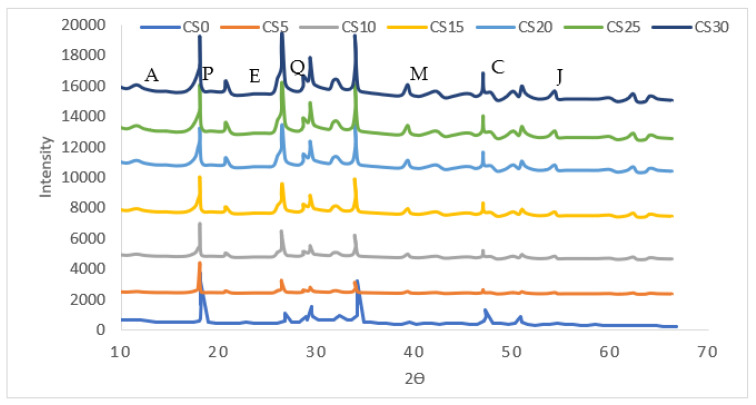
XRD A: aluminosilicate; E: ettrignite; M: mullite; P: portlandite; Q: quartz; C: calcite; and J: Jaffeite.

Figure 14 depicts the results of an XRD test that was conducted to analyze the impact of silica fume and cenosphere on concrete hydration. In most cases, CS was thought of as an indication of the hydration response of cement. According to the findings, the intensity of CS decreased significantly as the number of silica fume molecules in the sample increased. According to the findings of a plethora of other research, silica fume particles have the ability to speed up the process of hydration of cement and rapidly interact to CS to form an increased amount of CeSeH gels, which is seen in the studies of Rahmat et al. and Zemi et al. [68,69]. The fact that the peak of SiO_2_ did not grow along with the level of silica fume suggested that silica fume was largely engaged in the hydration process, which resulted in a substantial consumption of silica fume. XRD graph also revealed the presence of traces of ettrignite, aluminosilicate, mullite, portlandite, quartz, calcite, and Jaffeite. As observed in the micrograph, both the silica fume and the CS were successfully dispersed throughout the structure, and the bonding was not broken. A micrograph of the CS25 specimen can be appreciated in Figure 13g; as a result of the reaction between the pozzolanic compounds and the excess calcium that was present in the CS, the structure has been destroyed. The fogginess of the surface increased, and there was a significant increase in CS. A scan of CS30 taken with a scanning electron microscope revealed, as seen in Figure 13h, that the material’s microstructure had completely broken down, revealing heavier cracks. All of the observations made using the scanning electron microscope, which can be found in Figure 13a–g, show that the SEM Image of CS and its effect on the microstructural component of the cement concretes. In the beginning, the CS functioned as a filler; however, as the proportion of replacement increases, the CS reacted with the hydration elements, causing the structure to disintegrate and the formation of microstructural fissures. This had an effect on the concrete’s strength and performance. Patterns of distorted and fractured cement were produced by cement pastes that had a higher CS replacement rate. The interaction illustrates the separation of the CS and cement paste boundary layers, and the crack traversed these regions as it moved forward. This separation was brought about as a result of the CS not being able to adhere to the calcium hydroxides found in the paste. The fact that the vast majority of the cracks went through CS is evidence of the mechanism that dissipates energy. Furthermore, CS surfaces that were isolated from cement components exhibited a lower level of chemical reaction [6,8]. The deterioration of the concrete was aided in part by the presence of an excessive amount of alumina silicate in the CS. Because of the effect that these alumina and silicate had on the pozzolanic reaction, there were voids in the product zone of the pozzolanic reaction. The amount of mullite in the CS has also been linked to the fraction of CS that is being replaced. It is more likely to find traces of ettrignite in specimens that have a higher percentage of CS, and the frequency of portlandite decreases as the amount of CS in the rock increases. It has been observed that the CS undergoes a chemical reaction with the hydration product, and this reaction has been linked to the decline in portlandite production [18]. The result of this is the microstructure breaking down and becoming less stable. As a consequence of this, an excessive addition of CS may lead to a decrease in the quality of the cement concrete and a reduction in the mechanical performance. However, as the percentage of CS increases, the strength begins to decrease This is because the cement concrete containing CS replacement and silica fume functions admirably when combined with an adequate quantity of CS.

The research on the properties of concrete based on cenosphere and silica fume has important practical implications for the construction industry. The results could aid in the development of sustainable building materials that possess superior mechanical properties and reduced environmental impact. Cenosphere inclusion in lightweight concrete has generated significant interest due to its low density and high thermal conductivity. Integrating cenospheres in concrete can result in lighter structures, improved insulation, and increased durability. The resulting lightweight and strong structural elements could significantly lower the cost and environmental impact of construction projects. Ongoing studies on cenosphere concrete aim to enhance its mechanical properties, thermal conductivity, and durability to enable its practical implementation in various applications, such as building construction, transportation, and infrastructure. Consequently, this research carries significant implications for sustainable development and the construction industry. By replacing cement with industrial waste materials, such as cenospheres and silica fume, the demand for traditional raw materials could be reduced, resulting in lower production costs and a decreased carbon footprint. Moreover, the lightweight nature of cenosphere concrete can facilitate easier transportation and construction, lowering the weight of concrete structures. This study’s outcomes will help create high-performance structures with good strength and durability for concrete.

## 6. Conclusions

The impact of using constant silica fume and varying CS as a partial replacement for cement in light weight concrete was investigated. The study found that up to 30% replacement of CS could be used in LWC without compromising the strength, but beyond this point, the strength decreased due to increased voids and capillary channels. Water absorption was reduced in all specimens, with the lowest value recorded in 30% CS replacement due to the denser structure of silica fume and admixed LWC from the CS. Further research could investigate ways to mitigate this decrease in strength and improve the durability of CS in hostile environments.

The results of this study provide valuable insights into the use of industrial waste materials, such as CS and silica fume, as partial replacements for cement in lightweight concrete. These findings can be applied to the development of more sustainable building materials that offer superior mechanical properties and lower environmental impact.

The replacement of cement with cenospheres and constant replacement of silica fume in lightweight concrete significantly reduces the self-weight of concrete without compromising its overall strength. The addition of up to 30% cenosphere reduces the compressive and splitting tensile strength of the concrete, but the strength remains within acceptable limits for the design mix. However, excessive replacement of cement with cenosphere (30%) results in a fully degraded microstructure with visible cracks. The use of cenosphere and silica fume as partial replacements for cement in lightweight concrete can have practical applications in the construction industry, particularly for structures where weight reduction is a critical factor.

The results of this study also demonstrate the potential for using industrial waste materials, such as cenosphere and silica fume, to develop high-performance structures that offer good strength and durability to concrete. The use of these materials can reduce the demand for traditional raw materials, leading to lower production costs and reduced carbon footprint. The lightweight nature of cenosphere concrete can also lead to a reduction in the weight of concrete structures, resulting in easier transportation and reduced construction costs.

## 7. Future Studies

In this study on lightweight concrete (LWC) using constant silica fume (SF) and changing cenosphere (CS) for partial cement replacement, there are limitations and potential improvements through future investigations. One limitation is the lack of certain investigations, such as the long-term durability, resistance to environmental factors (freeze–thaw cycles) and chemical exposure of cenosphere concrete. Additionally, exploring the potential use of CS in combination with other lightweight aggregates, such as expanded polystyrene or perlite, could further enhance the properties of LWC. Finally, assessing the environmental impact of using CS as a replacement material, including its carbon footprint and potential for recycling, would be valuable for future research.

## Figures and Tables

**Table 1:** Mineral composition and chemical composition of cement.

Mineral Composition			Chemical Composition							
C_3_S	C_2_S	C_3_A	Si0_2_	Al_2_O_3_	Fe_2_O_3_	MgO	CaO	K_2_O	SO_3_	Na_2_O
52	22.1	8.75	20.4	6.55	3.56	1.75	65	0.54	0.42	0.25

**Table 2:** Chemical composition of fly ash.

Chemical Composition						
Si0_2_	Al_2_O_3_	Fe_2_O_3_	CaO	MgO	K_2_O	SO_3_
96.1	0.25	0.5	0.25	0.56	0.56	0.12

**Table 3:** Chemical composition of CS.

Si0_2_	Al_2_O_3_	Fe_2_O_3_	MgO	CaO	K_2_O	SO_3_	Na_2_O
69.4	23.12	3.1	0.8	1.04	0.3	1.2	0.02

**Table 4:** Physical Properties of CS.

Physical Property	Bulk Density kg/m3	Pycnometer Density kg/m3	Moisture %	Sinkers %	LOI %	pH	Color	Oil Absorption g/100 g
Value	475	812	0.1%	0.6%	0.41%	8	Grey	16

**Table 5:** Mix proportions of cenosphere concrete by weight.

Mix	Replacement %	Cement Kg/m^3^	Cenosphere Kg/m^3^	Fine Aggregate Kg/m^3^	Coarse Aggregate Kg/m^3^	W/c
CS	0	299.6	0	320	648	0.45
CS5	5	282.6	17	320	648	0.45
CS10	10	265.6	34	320	648	0.45
CS15	15	248.6	51	320	648	0.45
CS20	20	231.6	68	320	648	0.45
CS25	25	214.6	85	320	648	0.45
CS30	30	197.6	102	320	648	0.45

**Table 6:** Properties and strength of test specimens.

Mix	Slump	Density	Compressive Strength (MPa)			Split Tensile Strength (MPa)	Flexural Strength (MPa)	Water Absorption
	mm	Kg/m^3^	7 Days	14 Days	28 Days	28 Days	28 Days	%
CS	100	2452.5	19.43	31.50	36.38	3.31	4.51	1.81
CS5	95	2398.2	17.27	29.66	35.07	3.28	4.28	1.48
CS10	90	2222.4	16.02	27.62	33.18	3.15	4.12	1.42
CS15	80	2136.7	14.97	26.72	31.61	3.06	3.80	1.13
CS20	80	1908.9	12.21	23.94	29.72	2.93	3.54	1.27
CS25	70	1796.4	11.01	20.84	27.83	2.80	3.49	1.43
CS30	65	1548.9	9.52	19.36	26.60	2.72	3.33	1.28

## Data Availability

Not required.
